# Effects of pH, Total Soluble Solids, and Pectin Concentration on Color, Texture, Vitamin C, and Sensory Quality of Mango Fruit Bar

**DOI:** 10.1155/2023/6618300

**Published:** 2023-08-03

**Authors:** Ngoc Duc Vu, Van Muoi Nguyen, Thanh Truc Tran

**Affiliations:** ^1^Institute of Applied Technology and Sustainable Development, Nguyen Tat Thanh University, Ho Chi Minh City 700000, Vietnam; ^2^Faculty of Food and Environmental Engineering, Nguyen Tat Thanh University, Ho Chi Minh City 700000, Vietnam; ^3^Institute of Food and Biotechnology, Can Tho University, Can Tho City 94000, Vietnam; ^4^School of Graduate, Can Tho University, Can Tho City 94000, Vietnam

## Abstract

Mango purée is a byproduct of the current production processes (such as freeze-drying, dehydration) after the product shaping stage or grades II and III mangoes. Currently, fruit bar is a convenient and highly nutritious snack made from fruit. The objective of this study is to utilize mango byproduct in order to develop a fruit bar processing technology, which is based on evaluating the quality (color, break force, vitamin C content, and sensory) when varying the pH of mango purée and the concentration of added pectin. Additionally, total soluble solids (TSS) after blending at 80°C were also investigated. The increase in pH, pectin concentration of mango purée, and TSS after blending showed that vitamin C content in fruit bars tended to decrease. TSS results revealed that at TSS = 63°Bx, pH 3.3, and a pectin concentration of 1.3%, the product received a high rating of 6.3. Additionally, the vitamin C content of the product reached 7.82 mg/100 gDW. The results of this study are expected on the diversification of products from mango. Solving the situation that grades II and III mangoes are difficult to be commercialized and making the most of the byproduct mango flesh after certain production processes.

## 1. Introduction

Fruit bars are a kind of snack, typically made by pureeing fruit with starch and undergoing some dehydration process [1, 2]. This snack is known for their rectangular shapes, shiny surfaces, moderate hardness, and slightly chewy texture [3]. Fruit bars are rich in various nutrients, such as fiber, vitamins, minerals, energy, calcium, iron, and phosphorus. [4]. The specific nutritional content of these bars depends on the ingredients used. They are popular as snacks suitable for all ages, and their extended shelf life sets them apart from many other confectionery products. The low moisture content of the product leads to inhibition of microbial activity which is the main cause of product spoilage. However, the product still retains the appropriate toughness and hardness. Fruit bar products are formed on the need to consume nutrients and minerals from fruits. Currently, mango fruits are facing major spoilage problems during storage. It is not possible to consume more mango fruits within the end of the season, thus the seasonal fruits are processed to increase the shelf life. On the other hand, mangoes with significant defects are consumed domestically (in Vietnam) at low prices (0.11–0.68 USD/kg) in large quantities and are not traded on the international market [5]. Furthermore, after the specific shaping stage for the product, certain mango processing methods have resulted in a significant surplus of mango flesh. Simultaneously, there is a growing demand for a variety of mango-based products [6]. This is the driving force to promote the development of fruit products. Fruit bars are highly convenient as they provide the body with the nutrition of fruits at any time without the need for preparation [7]. Fruit bar products usually do not use preservatives, which are dehydrated to achieve low water activity, and the concentrated levels of antioxidant compounds and nutrients help prevent the growth of microorganisms and oxidation processes [7]. Therefore, fruit bar products has led to an increase in fruit consumption and has positively impacted human health. Large quantities of fresh fruit are processed, and there is limited spoilage of fresh fruit when stored for a long time. The fruit bar is made from a variety of fruits. However, fruit bar products cannot be processed from puréed fresh fruit without the participation of some suitable additives (pectin, maltodextrin, carboxyl methyl cellulose, and soluble starch etc.) [8]. In India, many types of fruit are used to create fruit bar products such as papaya, guava, jackfruit, and kiwi [9]. Some other reports also mention some ingredients commonly used to make fruit bars, such as apples, chiku, and jackfruit [10–12]. A previous mango fruit bar processing reported that cane or jaggery was added to the mango purée in a ratio of 1 : 2 or 1 : 4 and dried under the heat of the sun [2]. As the same time, the processing of fruit bars with ingredients including cane sugar, potassium metabisulphite, and mango purée, and the process of blending mangoes by hot air at 50–60°C from 18 to 22 hours. However, the result showed that the fruit bar was quite hard and not acceptable [2]. The fruit bar product from guava purée has improved in acceptable hardness. The product is dried by the convection drying method until the remaining moisture content reaches 14–15% [13].

Mango (*Mangifera indica* L.) is one of the plants that contain many nutrients and minerals (e.g., vitamin C at 36.4 mg/100 g, vitamin A at 1.082 IU) [14]. However, the shelf life of fresh mangoes is limited to only 30 days at 4–5°C, and it may be even shorter if the storage conditions are not optimal. This affected the nutritional quality of raw materials such as the degradation and oxidation of vitamin/polyphenol compounds. The prolonged storage process leads to uncontrolled spoilage, leading to losses for farmers and the national economy. In the past, measures to diversify mango products have been developed, such as mango jam [15], dried mango [16], canned mango juice [15], and rice paper [5], with the aim of meeting the diverse needs of consumers and adding value to mango fruits, improving regional and national economies. Some previous reports have revealed about the technologies for processing fruit bars from various fruits, such as guava [2], fig mango [17], and banana-papaya [18]. However, reports on the direct production formula of fruit bars from mango purée and the evaluation of their physical quality to fit the taste and sensory preferences of Vietnamese people have not been found. A previous report on mango bars was produced using mango powder/sliced mango peel without the use of preservatives or sweeteners. The study focused on evaluating the biological activities of the product, and the resulting product had an uneven surface [19]. A similar report focused on evaluating the nutritional composition and microorganisms in the product [20]. However, these studies did not use sugar, starch, or acid regulators in the processing, and the evaluation of product quality was limited in terms of texture, vitamin C content, color, etc. Moreover, sensory evaluation has not been assessed or has been assessed with a small number of representatives (10 panelists) and yielded average results.

This study is aimed at utilizing fresh grades II and III mangoes that did not meet export standards, along with the usable parts of mangoes damaged by bruising and overripe fruits (Codex Stan 184 : 1993). Furthermore, the byproduct mango flesh obtained from specific production processes such as freeze-drying and dehydration will be utilized for further processing purposes. During the processing, several factors were investigated, including pH, pectin concentration before blending process, and total soluble solids (TSS) after blending process. Various criteria were applied to monitor product quality, such as color (*L*^∗^, *a*^∗^, and *b*^∗^), break force, sensory evaluation, total ascorbic acid (vitamin C) (TAA), moisture content (MC), and water activity (*a*_*w*_). The findings of this study are expected to contribute to the diversification of mango products in the industry and reduce the wastage of fresh mangoes due to untimely consumption. Moreover, it provides a foundation for enhancing the value of mango fruits and minimizing the disposal of mango flesh resulting from certain production processes.

## 2. Material and Methods

### 2.1. Samples

Fresh grades II and III mangoes and usable parts of mangoes that are partially damaged by crushing, peeled, overripe, and byproduct mango flesh after certain production processes were collected in Can Tho city (coordinate 10°01′57^″^N, longitude 105°47′03^″^E). About 30 kg of raw material was pureed, metal canned, and frozen at -30°C; the center temperature of each box of mango purée is -18°C [21]. Mango purée was slowly defrosted at a temperature of 4–5°C before use. Some parameters are considered as input quality standards, such as TSS = 17.43 ± 0.42°Bx [22], pH 4.73 ± 0.33, yellow color (*L*^∗^ = 41.63; *a*^∗^ = 9.71; *b*^∗^ = 26.23), moisture content (MC = 83.93 ± 0.09%), and total acid (0.39 ± 0.04%).

### 2.2. Chemicals and Agents

Some chemicals and additives were used in this work, such as citric acid (E330), pectin (E440, degree of esterification = 50%), tapioca starch (≥ 85%), sugar (glucose, fructose, and maltose) purchased in Vietnam, 2,6-dichlorophenol indophenol (purity > 99.7%), L-ascorbic acid (purity 99%), HCl (purity 36.5%), oxalic acid (purity 98%), NaOH (purity > 98%), and phenolphthalein 1% (reagent) was purchased at Sigma-Aldrich.

### 2.3. Processing

**Figure 1:**
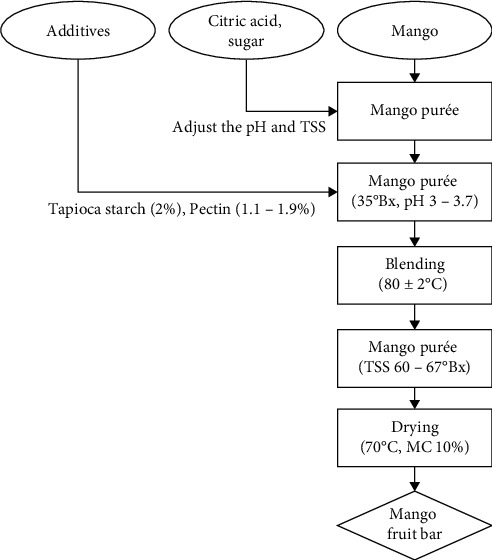
The process of production fruit bar from mango.

Mango purée was supplemented with sugar to adjust the initial total soluble solids to reach 35°Bx [23]. Tapioca starch was added at a concentration of 2% (*w*/*w*), and citric acid was used to adjust the pH of the mixture to 3–3.7 (pH/temperature bench meter HI2210-02, Romania). Pectin was then added to the mixture at a concentration of 1.1–1.9% (*w*/*w*). Next, the blending process was performed at 80 ± 2°C until TSS in the mango purée mixture achieve a range of 60–67°Bx [24] (ATAGO PR-101 Alpha 45°Brix, Japan). The mango purée mixture was filled into a mold measuring 30 *cm* *x* 60 *cm* *x* 2 cm, with a thickness of 1 cm, and it was convectively dried at a temperature of 70°C with a wind speed of 50 Hz until it reached a final moisture content of 10%. Color (*L*^∗^, *a*^∗^, and *b*^∗^), total ascorbic acid (TAA), breaking force, moisture content (MC), water activity (*a*_*w*_), and sensory value of fruit bars were tested at all processing conditions (Figure 1).

### 2.4. Determination of Moisture Content

The moisture analyzer (MB120, Ohaus, USA) was used to assist in determining the moisture content of fruit bars and fresh mango [25].

### 2.5. Determination of Activity Water

The Lab Touch-aw instrument (Novasina AG, Lachen, Switzerland) was used to assist in the determination of water activity (*a*_*w*_) [26, 27].

### 2.6. Determination of Color (*L*^∗^, *a*^∗^, and *b*^∗^)

The colorimeter device (CR-400/CR 410, Minolta, Japan) was used to assist in color determination which operates on the CIE *L*^∗^*a*^∗^*b*^∗^ color space. The *L*^∗^ value represents the luminance of the material with a range of 0 to 100 corresponding to the color range from black to white. The value +*a*^∗^ (+60) represents red, and −*a*^∗^ (-60) represents green. The value +*b*^∗^ (+60) represents yellow, and −*b*^∗^ (-60) represents blue. The three axes *L*^∗^, *a*^∗^, and *b*^∗^ combine into a three-dimensional color space. The machine was calibrated with white porcelain tiles (LO = 97.63; AO = 0.31; and BO = 4.63) before determining the color of the mango fruit bar [28].

### 2.7. Determination of Break Force (Hardness)

Break force is the force required to break the fruit bar's texture. It was determined based on the shear force of the Texture Analyzer TA_XT2i (Stable Micro System, England) with the D25 mm Dia Cylinder Aluminum probe. The measuring head is spherical, 2 cm in diameter. Compression speed is 2 mm/s; total force acting on the product is 25 kg; and break distance is 5 mm. The strength of the material is calculated based on the product of the impact force and the breaking distance. Break force was measured with 10 repetitions [29].

### 2.8. Determination of Total Ascorbic Acid

The titration method used to determine the total ascorbic acid (vitamin C) followed the principle of oxidizing vitamin C with a solution of 2,6-dichlorophenol-indophenol (DCPIP). The blue color of the DCPIP solution fades when it reacts with ascorbic acid. Excess DCPIP was added until a drop of the solution resulted in a light pink color that lasted for 30 seconds. Each 1 g of sample was extracted with 100 ml of distilled water. A mixture of 10 ml of the extracted solution and 1 ml of 0.04% HCl solution was vigorously shaken and titrated using a DCPIP solution. A control sample was prepared similarly, using 0.1 g of standard ascorbic acid instead of the 1 g sample extraction [30, 31].

### 2.9. Sensory Evaluation

Products were rated according to preference, based on the Hedonic scale with at least 60 panelists. Mango fruit bars must meet the following criteria: The mango fruit bars should exhibit a firm texture while maintaining a soft and flexible consistency, a brilliant reddish brown, a distinctive mango aroma, moderate sweetness, and balanced acidity in the product. The scale ranges from 1 to 9 [32].

In which, 9 = like extremely, 8 = like very much, 7 = like moderately, 6 = like slightly, 5 = neither like nor dislike, 4 = dislike slightly, 3 = dislike moderately, 2 = dislike very much, and 1 = dislike extremely [24].

### 2.10. Statistical Analysis

Statgraphics Centurion XV version 15.1.02 was used in this study with statistical significance (p < 0.05) [33].

## 3. Result and Discussion

### 3.1. Effect of the Blending Process on the Quality of the Fruit Bar

**Figure 2:**
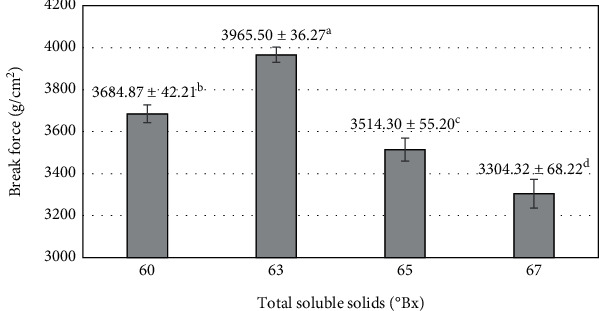
The influence of total soluble solids (TSS) after the blending process on the break force of mango fruit bar (hardness). Different letters (a–d) represent statistically significant differences (*p* < 0.05).

Hardness is one of the important factors that directly affects the sensory value of the product and the consumer experience. Hardness can be understood as the force required to break the shape [34]. The blending process at 80°C increased the TSS of the mango purée, reaching 60–67°Bx. This increase is associated with the blending time, as longer durations lead to greater water evaporation. The blending time required to achieve different levels of TSS significantly affects the breaking force of the fruit bars (*p* < 0.05). The highest value was achieved at 63°Bx after the blending process (3965.5 ± 36.27 g/cm^2^) and tended to decrease as TSS continued to increase to 67°Bx (3304.32 ± 68.22 g/cm^2^). However, increasing TSS from 60 to 63°Bx showed a significant increase in the break force (*p* < 0.05) (Figure 2). This is explained by the gelling ability of pectin and starch in an acidic medium [35]. The combination of short pectin chains and water results in the formation of an extensive network of pectin chains [36]. Simultaneously, water is absorbed into the pectin network, creating a colloidal system comprising a solid dispersion medium and a liquid dispersion. The texture becomes softer and more easily deformable as the pectin network absorbs more water. The blending process duration is directly proportional to the increase in TSS and inversely proportional to the MC in the material. TSS increased from 60 to 63°Bx is the period of high MC which is responsible for the soft texture of the fruit bar. Break force reached its highest value at 63°Bx and tended to decrease as TSS continued to increase. MC in raw materials is too low, and the colloidal ability of pectin is reduced due to the limited linkage between short pectin chains [37]. The elevated MC in the raw materials enhances the pectin chains' ability to form links, resulting in elongation of the pectin chain. This elongation caused a reduction in the strength of the pectin chain, making the product's texture more prone to breakage when subjected to external forces or chemical reactions. The results were similar to a previous report on the reduction of shear force after dewatering the fruit bar product from blended aonla-guava [38].

**Figure 3:**
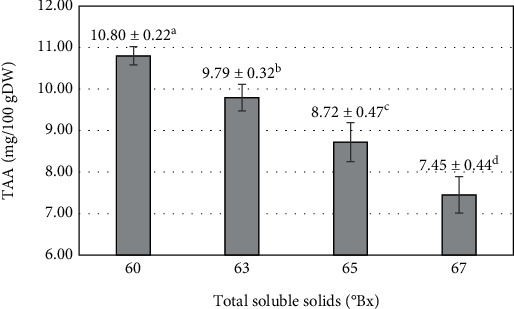
The influence of total soluble solids after the blending process on the total ascorbic acid (TAA) of mango fruit bar. Different letters (a–d) represent statistically significant differences (*p* < 0.05).

Ascorbic acid has been recognized as an essential nutrient that cannot be synthesized by the body [39]. However, it is sensitive to both temperature and light. Moreover, the duration of the processing time is a factor that can accelerate the degradation of ascorbic acid [40]. Subsequent to the blending process, the TSS reached 60°Bx, corresponding to a TAA in the purée mixture of 10.8 ± 0.22 mg/100 gDW. As the blending time continued to increase, the TSS of the purée mixture exhibited a tendency to rise, while the TAA decreased inversely (Figure 3). At the point when the purée mixture reached 67°Bx, the TAA reached its lowest value at 7.45 ± 0.44 mg/100gDW. The continuous degradation of TAA can be attributed to the duration of the heating process during blending. As the TSS of the product increases, the blending time at a temperature of 80°C also increases accordingly. Prolonged exposure to this elevated temperature significantly impacts the TAA, leading to its degradation. The influence of temperature on vitamin C has also been reported through the heat processing of spinach and fenugreek [41]. A previous study indicated that vitamin C in fruit bars made from kiwi, spinach, and bina leaves was significantly reduced during the blending process at high temperatures [42]. The production process of guava fruit bars through convective drying at 65°C resulted in a decrease of approximately 24.5% in vitamin C content compared to fresh guava [43]. The reduction of vitamin C in apple and strawberry fruit bars was also revealed during the drying process at 50°C for 25 minutes [44].

Water had the ability to evaporate under normal conditions. Temperature acted as a catalyst that accelerates the evaporation of water. TSS represented the number of soluble solids in a solution. The higher the TSS value, the higher the TSS ratio in the solution. During the prolonged blending process, the TSS of the mixture increased (Table 1). Simultaneously, the blending time was prolonged, resulting in an increase in the amount of water evaporated. The MC of the mixture tended to decrease as the TSS increased from 60 to 67°Bx (12.57 ± 0.2%) [44]. In an environment with high-temperature conditions, the water molecules moved faster and more chaotically, leading to the diffusion of water vapor into the air. The blending process was directly proportional to the increase in TSS and inversely proportional to the MC in the material. Consequently, as the TSS increased, the evaporation capacity of water increased [45]. The results were similar to a report on the moisture reduction of sapota fruit bars when subjected to heat (91°C) for 150 minutes to increase the TSS of the fruit bars to 75 ± 3°Bx [46]. Another report revealed that strawberry fruit bars treated at 50°C reduced the moisture content to 16% after 6.75 hours. Under the same heat treatment conditions, guava fruit bars took about 5 hours to achieve a similar moisture content [47]. An inverse correlation between TSS and heating time has also been reported in the dehydration process of tomatoes [48]. In addition to fruit bars, prolonged high-temperature processing also increases the evaporation capacity of water in beets [49].

Water activity is a measure of the free water content in food. It is considered an important parameter directly related to the growth of bacteria, mold, and other microorganisms in food products. Environments with higher water activity create favorable conditions for the development of harmful microorganisms, causing spoilage, fly infestation, and deterioration of product quality [50]. After the blending process, the amount of free water was vaporized from the material, resulting in a decrease in water activity (*p* < 0.05). The *a*_*w*_ value reached the highest value again at TSS 60°Bx (0.69 ± 0.02) and tended to decrease as TSS increased to 67°Bx (0.57 ± 0.02). A positive correlation between *a*_*w*_ and moisture content has also been reported previously in the dehydration process of beets [49], honey [27], and flowers [51]. A report on the reduction of water activity in apple fruit bars achieved a range of 0.46–0.48 after a water separation process using heat [7]. In this study, the blending process involved the use of heat to dissolve sugar and citric acid. However, the adverse effect of the blending process on water activity was also observed. Nevertheless, at the water activity levels obtained after blending, there is still a risk of creating a favorable environment for the growth of microorganisms. Therefore, a drying process is necessary to further reduce the water activity in the product and inhibit the activity of harmful microorganisms.

Color is one of the important factors in determining the acceptability of food products for consumers [52]. During the blending process, the time required for blending was proportional to the product's TSS. The higher the TSS of the product, the longer the blending time. The blending temperature and time were the main factors that affected the color change of the product during processing and had a significant influence on the product's *L*^∗^ lightness (*p* < 0.05) (Table 2). The blending time was extended until the TSS of the mixture reached 60–63°Bx, resulting in the highest lightness with an *L*^∗^ value of 38.26 ± 0.27. As the time was further extended to reach a TSS of 65–67°Bx, the *L*^∗^ value tended to decrease to 36.98 ± 0.17. This decline can be explained by the effect of temperature on the sugar content of the raw material, which caused the caramelization reactions and led to a decrease in product lightness, resulting in a darker color. Additionally, the heat sensitivity and oxidation of the diene chains in carotenoids also contributed to the decrease in the *L*^∗^ value of the product [45]. However, no significant changes (*p* > 0.05) were observed in the yellow color (*b*^∗^) of the product, which remained stable at 12.23 ± 0.33. A similar report showed a reduction in lightness (*L*^∗^) from 30.40 ± 1.20 to 25.70 ± 0.78 during the water removal process in sapota fruit bars using heat [46]. Previous studies on sapodilla fruit bars also revealed a similar decrease in lightness (*L*^∗^) with prolonged heat processing until reaching a moisture content of 19.9% [45]. In addition to color changes in fruit bars, prolonged water removal processes in certain products like bananas [53], apples, bananas, potatoes, and carrots have also been found to cause a decrease in lightness [54].

**Figure 4:**
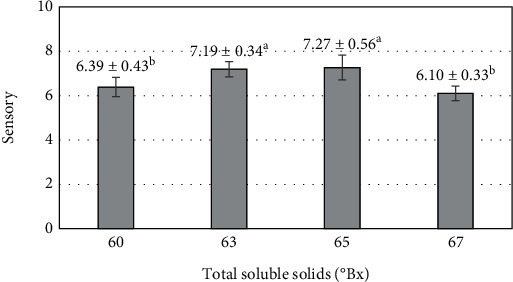
Effect of total soluble solids after the blending process on sensory value of mango fruit bar. Different letters (a–b) represent statistically significant differences (*p* < 0.05).

The overall acceptability of the mango fruit bars was evaluated by 60 panelists on a 9-point scale based on criteria such as texture, taste, and color of the product. The overall evaluations of the different survey levels indicated that they were generally accepted by the majority of the panelists. The highest ratings (7.19–7.27 points) were found for the fruit bars made with mango purée that achieved TSS levels of 63–65°Bx, which were considered the most balanced products within the survey range (Figure 4). Comments from the panelists indicated that the sweetness, acidity, and color of the product were comparable. However, the lightness of the fruit bars made from mango purée with a TSS of 67°Bx was perceived to be lower. On the other hand, the aroma of the product made with mango purée at a TSS of 60°Bx and dried to a moisture content of 10% was described as better compared to the product blended to a TSS of 67°Bx. A previous report revealed that a TSS of 63°Bx was suitable for the production of papaya jam bars [55].

### 3.2. Effect of pH on the Quality of the Fruit Bar

**Figure 5:**
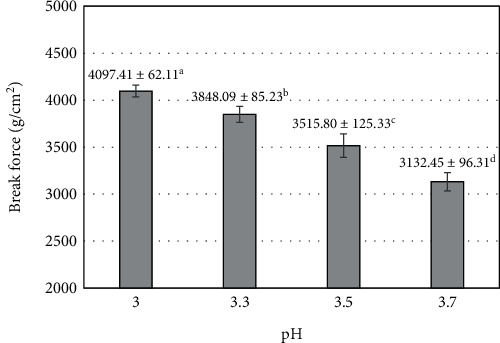
The influence of pH (mango purée) on the break force of mango fruit bar. Different letters (a–d) represent statistically significant differences (*p* < 0.05).

The hardness of food products made from fruit purée is influenced by various factors, including enzyme activity in raw materials, pectin, calcium content, gelling ability, and dehydration of the raw material mixture. The break force of the product was found to be significantly affected by the pH of mango purée (*p* < 0.05). At pH 3, the highest break force value was reached at 4097.41 ± 62.11 (g/cm^2^) and significantly decreased when increasing the pH to 3.7 (3132.45 ± 96.31 g/cm^2^) (Figure 5). The decrease in break force as the pH value increases can be attributed to the activity of several enzymes, including endopolygalacturonase activity. Endopolygalacturonase is known to be active within the pH range of 4.5 to 5.0 [56]. The closer the pH adjustment is to the optimal active pH range of endopolygalacturonase, the higher the enzyme activity within the cell. A previous report revealed that increased enzyme activity leads to damage in fruit texture. At the same time, a positive correlation between pectin solubility in water and fruit hardness was also revealed [57]. On the other hand, pectin has a better solubility in an acidic environment and contributes to cell binding. As the pH increases, the environment becomes more alkaline, causing pectin to soluble less effectively. The solubilization of pectin leads to the formation of a large network of pectin chains by fusing short pectin chains together. Concurrently, water absorption occurs within this pectin network, resulting in the formation of a colloidal system. During the water removal process (blending) from the pectin material, the network contracts and becomes intertwines, leading to a harder texture of the fruit bar [58].

**Figure 6:**
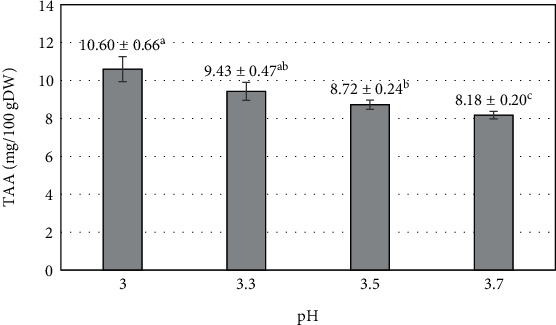
The influence of pH (mango purée) on the total ascorbic acid (TAA) of mango fruit bar. Different letters (a–c) represent statistically significant differences (*p* < 0.05).

Citric acid is a compound that has antioxidant properties and indirectly protects vitamin C from oxidation by creating an increasingly acidic environment and inhibiting the activity of enzymes [59]. The enzymatic activity during processing significantly reduced vitamin C compared to fresh mango (19.96 ± 1.70 mg/100gDW). The best vitamin C protection efficiency was observed at pH 3, with remaining TAA reaching 10.60 ± 0.66 mg/100 gDW. As the pH increased from 3 to 3.7, TAA tended to decrease and reached the lowest value at pH 3.7 (8.18 ± 0.20 mg/100 gDW) (Figure 6). This decrease can be explained by several factors, such as the better stability of TAA in an increasingly acidic environment and reduced its ability to be decomposed by light temperatures [60]. Additionally, the optimum pH range for PPO enzyme activity has been previously reported to be between 3 and 9, with pH 6–6.5 being optimal for PPO activity [61]. Increasing the pH closer to the optimal range of PPO activity increases the level of PPO oxidative activity and the oxidation process of vitamin C [31]. On the other hand, the pH change from 3 to 3.7 is related to the amount of added citric acid. Lower pH requires a larger amount of added citric acid to reduce the pH of mango purée. At pH 3.7, the low amount of added citric acid is insufficient to affect the oxidative agents (PPO) and prevent them from losing their oxidative properties. This results in reduced effectiveness in preventing the impact of oxidative agents on vitamin C. Consequently, under the influence of oxidative agents, vitamin C is reduced more in comparison to fruit bars processed from purée with a pH 3. A previous report mentioned the enhancement of the antioxidant capacity of Madagascar periwinkle roots by simultaneous supplementation of oxalic acid, citric acid, and malic acid [62].

Customers frequently rely on color as a criterion to evaluate product quality, even without physically using the product [63]. The color of a material is determined by various pigments such as chlorophyll, carotenoids, anthocyanins, and myoglobin. However, these pigments can be affected by oxidative processes. Carotenoids, for instance, are a group of pigments primarily present in mangoes and are prone to chemical degradation during processing [64–67]. Fruit bars made from mango purée with increasing pH levels from 3 to 3.7 significantly affected the *L*^∗^ and *b*^∗^ values of the product (*p* < 0.05). However, the change in the *a*^∗^ value was not significant and remained stable at around 30.92–31.16 (*p* > 0.05) (Table 3). At pH 3, the highest *L*^∗^ value was observed with 41.14 ± 0.61, and it decreased gradually as the pH increased to 3.7 (*L*^∗^ = 36.55 ± 0.60). The color of the product became darker as the pH of the mango purée increased. This variation is associated with the presence of added citric acid and the degradation process of polyphenolic compounds. The polyphenolic compounds are degraded and produce melanin, which contributes to the brown coloration [68]. At low pH, corresponding to higher levels of citric acid added to the mango purée, this inhibited the activity of polyphenol oxidase (PPO). The degradation of polyphenolic compounds was also limited. Therefore, at pH 3, the color appeared brighter. However, the effectiveness of citric acid in color retention is only a small part of the many factors contributing to the degradation of polyphenolic compounds. A previous study revealed an increase in citric acid concentration in the pre-processing of water spinach, resulting in increased lightness and color of the product [69]. Similar results were observed in the application of citric acid to reduce color changes in cabbage [70]. The addition of citric acid to a membrane coating mixture also preserved the vibrant red color of pomegranate fruits [71].

**Figure 7:**
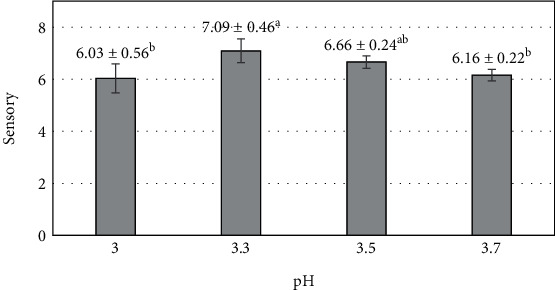
The influence of pH (mango purée) on the sensory value of mango fruit bar. Different letters (a–d) represent statistically significant differences (*p* < 0.05).

Citric acid has been used to adjust the pH of the purée. The lower the pH, the higher the amount of citric acid added. Fruit bars made from mango purée with a pH ranging from 3 to 3.7 had a significant statistical impact on the overall acceptability by the 60 panelists (*p* < 0.05). Fruit bars made from mango purée with pH 3 were evaluated as having an unbalanced taste between the sweetness of the sugar and the sourness of the citric acid, with the sour taste predominating and causing discomfort when consuming the product (6.03 ± 0.56 points) (Figure 7). Increasing the pH to 3.3 resulted in a reduction in sourness and a better balance between the sweetness of the sugar and the sourness of the citric acid. At the same time, the texture was not excessively firm or soft. Comments from the panelists indicated that as the pH increased from 3.5 to 3.7, the sourness continued to decrease, and the sweetness of the sugar became dominant, creating a perception of excessive sweetness. A general consensus among most panelists was that a pH of 3.3 was suitable for commercializing the product (7.09 ± 0.46). A previous report on guava fruit bars revealed that increasing the acid content in the guava purée mixture decreased consumer acceptance, with the lowest score (4.79 points) observed in Rajendranagar [72].

### 3.3. Effect of Pectin Concentration on the Quality of the Fruit Bar

**Figure 8:**
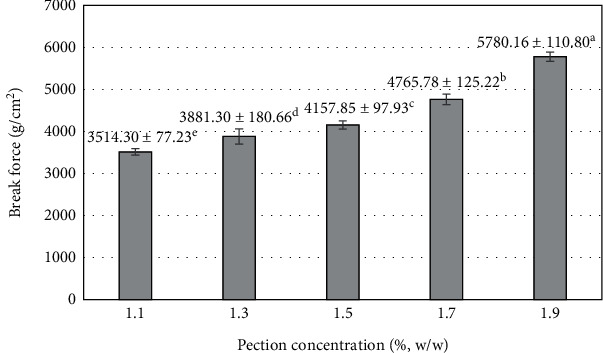
The influence of pectin concentration on the break force of mango fruit bar. Different letters (a–e) represent statistically significant differences (*p* < 0.05).

Pectin readily gels in aqueous media to form a 3D pectin network. Pectin-long chains containing galacturonic acid residues were interrupted by chain branching and the incorporation of rhamnose. Even at low concentrations, pectin readily forms gels in the presence of water. Pectin is used as a thickener and stabilizer in various food industries [73]. A pectin concentration investigation is necessary to meet the economic efficiency and sensory value of the product. The results of the investigation showed that pectin concentration had a significant effect on break force (Figure 8). The break force continuously increased with the addition of pectin concentration to the blended mixture. The highest break force was at a pectin concentration of 1.9% (5780.16 ± 110.8 g/cm^2^), and break force was found to tend to increase with an additional increase in pectin concentration. The higher the pectin content in the aqueous medium, the stronger the binding capacity and the number of bonds between the pectin short chains to form a 3-dimensional network. During the drying process of the product, the pectin chains shrink and intertwine, increasing the durability of the texture. Hardness increased when adding pectin from 1 to 3% during the processing of sapodilla fruit bars [45]. Similarly, the production of kinnow bars with the addition of pectin at concentrations ranging from 1 to 4% significantly increased hardness [74]. A similar result was observed in the blending process of potato starch with pectin from 1 to 4%, which increased hardness from 205.55 to 244.35 g/cm^2^, but when pectin was added at 5%, hardness decreased [75].

**Figure 9:**
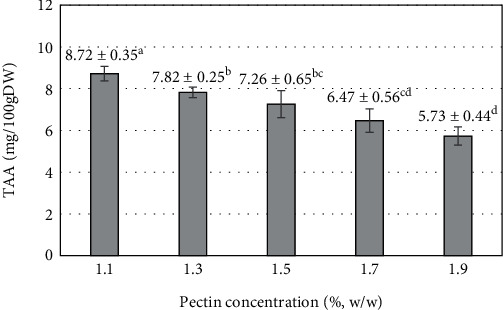
The influence of pectin concentration on the total ascorbic acid (TAA) of mango fruit bar. Different letters (a–d) represent statistically significant differences (*p* < 0.05).

Pectin is a natural fiber found in most plants. The addition of pectin increased the fiber content of the blended mixture. The results of the pectin concentration investigation added to the blend from 1.1 to 1.9% showed that there was a statistically significant influence on TAA (*p* < 0.05). In pectin concentration supplemented with 1.1%, the highest TAA value (8.72 ± 0.35 mg/100 gDW) was obtained (Figure 9). Continuing to increase the pectin concentration in the blended mixture, TAA tended to decrease significantly. Increasing pectin concentration decreased the density distribution of L-ascorbic acid molecules per unit analyzed. A similar result was found in a previous report where increasing the addition of pectin from 1.0 to 1.5% resulted in a decrease in vitamin C content from 21.23 mg/100 g to 20.50 mg/100 g [76].

**Figure 10:**
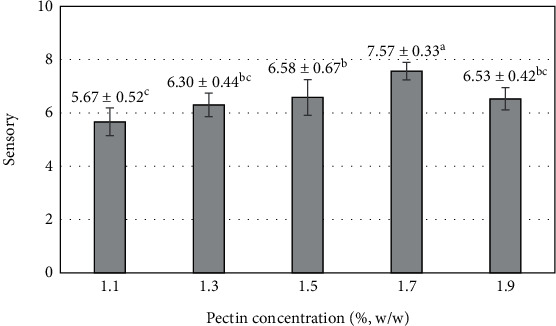
The influence of pectin concentration on the sensory value of mango fruit bar. Different letters (a–c) represent statistically significant differences (*p* < 0.05).

The sensory factor is one of the important indicators in the trade of products. The more pectin concentration is added to the blending process, the more it is appreciated by consumers. On pectin concentration, 1.7% was reached, which was highly appreciated by most consumers with a sweet taste, a mildly sweet taste, and a product with a moderate chewy texture. The added pectin concentration did not lose too much of the mango flavor (7.57 ± 0.33) (Figure 10). Pectin concentration was added from 1.1 to 1.7% with increasing consumer preference, products described as sweet and sour, and tended to increase the sense of harmony between sour, sweet, and distinctive flavors characteristic of mango. However, the increase in pectin concentration added to the blend reached 1.9%, and consumers tended to decrease their preference for the product. At a pectin concentration reaching 1.9%, the characteristic flavor of mango and sour taste rated by the majority of consumers was significantly reduced. A report on pineapple leather snack showed a decrease in overall liking of the product when the addition of pectin to the purée was increased [77]. Increasing the pectin content from 0 to 1.5% at pH 3.3 resulted in a reduced consumer acceptance of mango jam, as reported in a previous study [76].

## 4. Conclusion

This study has successfully evaluated the effects of TSS, pH, and pectin concentration on the quality of fruit bar products (texture, color, vitamin C, and sensory quality). The obtained results showed that in TSS after the blending process, it reached 63°Bx for a product with stable texture and suitable color and received high praise from most consumers. The mixture of mango purée and additives was adjusted to a pH value of 3.3 and 1.3% pectin with high efficiency in terms of texture, organoleptic quality, color, and retained TAA in the product. The results of this study are the basis for the development of fruit bar products from fresh grades II and III mango materials. Improve the economic efficiency of the mango growing area and diversify products from this raw material to meet the consumption needs of today's customers.

## Figures and Tables

**Table 1:** Effect of total soluble solids after blending process on the moisture content and water activity (*a*_*w*_).

TSS (°Bx)	MC (%)	*a*_*w*_
60	14.67 ± 0.17^a^	0.69 ± 0.02^a^
63	14.20 ± 0.21^b^	0.65 ± 0.01^b^
65	13.67 ± 0.14^c^	0.61 ± 0.01^c^
67	12.57 ± 0.20^d^	0.57 ± 0.02^d^

Noted: different letters (a–d) in same column represent statistically significant differences (*p* < 0.05).

**Table 2:** Effect of total soluble solids after the blending process on color (*L*^∗^, *a*^∗^, and *b*^∗^) of mango fruit bar.

TSS (°Bx)	*L* ^∗^	*a* ^∗^	*b* ^∗^	Color
60	38.26 ± 0.27^a^	31.12 ± 0.31^a^	12.23 ± 0.33^a^	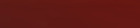
63	38.06 ± 0.22^a^	31.22 ± 0.40^a^	12.35 ± 0.31^a^	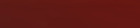
65	37.31 ± 0.12^b^	31.52 ± 0.30^a^	12.42 ± 0.42^a^	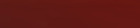
67	36.98 ± 0.17^b^	31.24 ± 0.44^a^	11.91 ± 0.43^a^	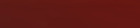

Noted: different letters (a–b) in same column represent statistically significant differences (*p* < 0.05).

**Table 3:** The influence of pH (mango purée) on the color (*L*^∗^, *a*^∗^, and *b*^∗^) of mango fruit bar.

pH	*L* ^∗^	*a* ^∗^	*b* ^∗^	Color
3	41.14 ± 0.61^a^	30.92 ± 0.26^a^	14.54 ± 0.52^a^	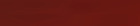
3.3	38.06 ± 0.22^b^	31.22 ± 0.40^a^	12.35 ± 0.31^b^	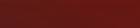
3.5	37.98 ± 0.54^bc^	31.02 ± 0.56^a^	12.71 ± 0.41^b^	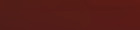
3.7	36.55 ± 0.60^c^	31.16 ± 0.37^a^	11.64 ± 0.21^c^	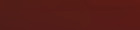

Noted: different letters (a–c) in same column represent statistically significant differences (*p* < 0.05).

## Data Availability

All data available in this paper.
